# Stability of sensor-based gait parameters reassessed after a period of one year in people with multiple sclerosis

**DOI:** 10.1186/s12883-023-03168-9

**Published:** 2023-03-24

**Authors:** Roy Müller, Daniel Hamacher, Philipp M. Keune, Patrick Oschmann

**Affiliations:** 1 Department of Neurology, Medical Campus Upper Frankonia, Klinikum Bayreuth GmbH, Bayreuth, Germany; 2 Department of Sports Science, Friedrich Schiller University Jena, Jena, Germany; 3 Department of Cognition, Emotion and Neuropsychology, Otto-Friedrich-University, Bamberg, Germany

**Keywords:** Gait, Reliability, Inertial sensors, MS, 6-min walk, EDSS

## Abstract

**Background:**

Currently, there are several studies showing that wearable inertial sensors are highly sensitive in the detection of gait disturbances in people with multiple sclerosis (PwMS), showing excellent reliability within one or 7–14 days. However, it is not known how stable these gait parameters remain over a longer period of time. This is surprising, because many treatments last longer than two weeks. Thus, the purpose of the current study was to examine gait parameters obtained by means of wearable inertial sensors during a 6-min walk and to reassess these parameters after a period of one year.

**Methods:**

Fifty PwMS (without a relapse or a recent change in the Expanded Disability Status Scale (EDSS) or treatment) and 20 healthy participants were examined at two assessment points (interval between assessments: 14.4 ± 6.6 months). At each assessment point, all participants had to complete a 6-min walking test, an observer-rater test (Berg Balance Scale, BBS) and a Timed-up and Go Test (TUG). To measure mean gait parameters (i.e. walking speed, stride length, stride time, the duration of the stance and swing phase and minimum toe-to-floor distance), as well as the intraindividual standard deviation of each mean gait parameter, wearable inertial sensors were utilized.

**Results:**

We found that even after one year all mean gait parameters showed excellent Intraclass Correlation Coefficients (ICC between 0.75 and 0.95) in PwMS. Looking at MS subgroups, the ICCs were slightly higher in MS subgroup 2 (EDSS 2.0–5.0) than those in MS subgroup 1 (EDSS 0.0–1.5) and healthy controls. Compared to the mean gait parameters, parameters of gait variability showed only good-to-fair ICC values in PwMS. Concerning BBS and TUG, the ICC values after one year were close to the ICC values of the measured mean gait parameters.

**Conclusions:**

Due to the excellent stability of mean gait parameters after one year, these sensor-based gait parameters can be identified as clinically relevant markers to evaluate treatment effects over a longer (several months) period of time in MS.

**Supplementary Information:**

The online version contains supplementary material available at 10.1186/s12883-023-03168-9.

## Background

Multiple sclerosis (MS) is a progressive chronic inflammatory disease with a variety of clinical symptoms that results in multiple neurological deficits with a major effect on balance control and walking ability (e.g., [1–9]). Moreover, around 40% of people with MS (PwMS) report walking problems that unfavorably affect their quality of life [10]. In order to improve PwMS's quality of life, early detection of gait deficits is crucial.

Currently, a few studies explored the potential value of wearable inertial sensors in the detection of gait deficits in PwMS (e.g., [8, 9, 11, 12]). This is striking, as the sensors could potentially be utilized to conduct automatic and standardized assessments to objectively identify walking deficits and monitor walking performance in PwMS [13]. However, there are several studies showing that wearable inertial sensors are highly sensitive in the detection of gait disturbances in MS, even in early MS, where global scales such as the Expanded Disability Status Scale (EDSS) do not provide specific clinical information about deviations in gait behavior (e.g., [8, 9, 11, 12]). Even though the EDSS remains the most common measure of functional disability in MS, it only provides relatively superficial information on walking capacity. For example, on its scale from 1.0 to 10, a value of 4.0 implies that walking of at least 500 m without support or rest is possible, which then decreases with higher EDSS values [14].

Additionally, wearable inertial sensors show good reliability within one or 7–14 days during short (e.g. 25-foot walk, 10-m test) and prolonged distances (e.g. 2-min and 6-min walk test). For example, Flachenecker et al. [12] investigated the correlation coefficient between consecutive 25-foot walking tests (two times at a self-selected speed, followed by two times at a speed as fast as possible) in 102 PwMS and 22 healthy controls and found high reliability (r = 0.9) for the mean gait parameters for both self-selected and fast walking speeds. In another study, 57 PwMS and 24 healthy controls were asked to walk back and forth a 10-m distance for six minutes at their comfortable speed [11]. The authors repeated the assessment on a second visit, which was held 7–14 days after the first one and found that the higher the EDSS score, the more the reliability was increased. These results obtained by means of wearable inertial sensors are comparable to previously reported test–retest analyses of a 6-min walk using a stop-watch [3] or an electronic walkway [15].

The studies mentioned above show excellent reliability within one or 7–14 days. However, it is not known how stable these objective sensor-based gait parameters remain over a longer period of time. We hypothesized that gait analysis after a longer period of time would reveal changes in gait parameters, especially in more disabled PwMS. Thus, the purpose of the current study was to examine gait parameters obtained by means of wearable inertial sensors during a 6-min walk and to reassess these parameters after a period of one year in PwMS.

## Methods

### Participants

Fifty PwMS and 20 healthy participants were recruited sequentially in the outpatient clinic of the Department of Neurology, Klinikum Bayreuth GmbH, Medical Campus Upper Frankonia, Germany (Table 1). PwMS were eligible to participate in case of a verified MS diagnosis [16], stable symptoms between both assessment points performed one year apart (i.e. no relapse and no treatment or EDSS change), an age between 18–65 years and the ability to walk without a walking aid for six minutes. PwMS were not included in case of a relapse or a treatment or EDSS change between both assessment points. All participants provided written informed consent. The study was approved by the ethical review board of the Friedrich Schiller University Jena, Germany (2018–1221-BO) and was in accordance with the Declaration of Helsinki.

**Table 1:** Demographical and clinical characteristics of the sample

		PwMS	Healthy controls
Sex [f/m]	MS	27/17	
	subgroup 1	12/8	11/9
	subgroup 2	15/9	
Age [years]	MS	41.5 ± 11.5*	
	subgroup 1	35.6 ± 10.4	35.0 ± 8.7
	subgroup 2	46.4 ± 10.2*	
Height [cm]	MS	172.5 ± 9.6	
	subgroup 1	172.5 ± 10.4	174.0 ± 8.4
	subgroup 2	172.5 ± 11.0	
Weight [kg]	MS	77.1 ± 18.5	
	subgroup 1	74.3 ± 14.9	71.4 ± 11.3
	subgroup 2	79.3 ± 20.9	
EDSS	MS	2.1 ± 1.2	
	subgroup 1	1.1 ± 0.4	n.a
	subgroup 2	3.0 ± 0.8	
Subtype of MS	MS	RRMS = 41, SPMS = 3	
	subgroup 1	RRMS = 20, SPMS = 0	n.a
	subgroup 2	RRMS = 21, SPMS = 3	

Values of age, height, weight and Expanded Disability Status Scale (EDSS) are expressed as mean ± standard deviation. Significant differences of PwMS (EDSS 0.0–5.0), MS subgroup 1 (EDSS 0.0–1.5) and MS subgroup 2 (EDSS 2.0–5.0) from healthy controls are indicated with ‘*’ (*p* < 0.05). MS subgroups were established using a median split based on EDSS scores

*MS *Multiple Sclerosis, *PwMS *People with MS, *RRMS *Relapsing–remitting MS, *SPMS *Secondary progressive MS

### Measurements

Participants were examined at two assessment points during routine clinical appointments in the Klinikum Bayreuth GmbH, Department of Neurology (interval between assessments: 14.4 ± 6.6 months). Out of the 50 PwMS, six experienced a relapse between assessments. Since a relapse potentially affects gait, and since six participants are too less to be analyzed as a subgroup, the datasets of these participants were excluded from the further analysis focusing on stability. EDSS values in PwMS remained identical across the two assessments.

At each assessment point, both PwMS and healthy participants had to complete a walking test that required them to cover a distance of 25 feet repeatedly throughout a period of six minutes as enduring and fast as possible (6-min walk). A cone was placed three feet away from each endpoint of the 25-foot distance and participants circled the cones to make their turn back toward the 25-foot distance [6, 8, 9]. To measure gait parameters (i.e. walking speed, stride length, stride duration, the duration of the stance and swing phase as well as the minimum toe-to-floor distance), wearable inertial sensors were utilized (MTw2, Xsens Technologies B.V.; sampling rate: 100 Hz). The sensors were attached to the forefoot of the participants’ dominant leg (i.e., the foot they would take to kick a ball; [9]). In addition to the walking test and gait parameter measures, an observer-rater test (Berg Balance Scale, BBS, [17]) and Timed-up and Go Test (TUG, [18]) were administered.

### Data processing

To exclude effects of acceleration and deceleration the first and the last 25 feet distances, as well as the first and the last 2.5 m of each distance between the cones were excluded from the following analysis [8, 9]. We used a validated algorithm [19] to calculate mean gait parameters (i.e. walking speed, stride length, stride time, the duration of the stance, swing phase and minimum toe-to-floor distance), as well as the intraindividual standard deviation of each mean gait parameter as gait variability measures. Heel strikes and toe-off events were identified based on local minima of the angular velocity of the foot in the sagittal plane [20].

### Statistical analysis

Statistical analyses were performed with SPSS 20 (Chicago, IL, USA). To test normality of distributions, Kolmogorov–Smirnov tests were implemented for all gait parameters (mean, standard deviation (SD)). Participant characteristics were compared using Pearson’s Chi-square for gender and independent t-Tests for age, height, weight and EDSS scores, separately for healthy controls, PwMS and both MS subgroups. MS subgroups (subgroup 1: EDSS 0.0–1.5, subgroup 2: EDSS 2.0–5.0) were established using a median split based on EDSS scores (e.g., [5]). Differences in mean gait parameters and gait variability (SD) between PwMS and healthy participants were assessed by a one-way between-subjects analysis of variance (factor GROUP with four levels: healthy controls, PwMS, MS subgroup 1, and MS sub-group 2) with Bonferroni post-hoc analysis. Stability was assessed using the calculation of Intraclass Correlation Coefficients (ICCs) with an ICC(2,1) model and 95% confidence intervals [21]. ICC values were interpreted as follows: > 0.75 was excellent, 0.60–0.74 was good, 0.40–0.59 was fair, < 0.40 was poor [22]. Furthermore, Bland–Altman analysis/ plots with 95% Limits of Agreement (LoA) for each measure were performed/ produced to assess the agreement of gait measures obtained in the two visits [23]. An alpha level of 0.05 was used for all statistical tests.

## Results

### Stability of mean gait parameters

**Fig. 1:**
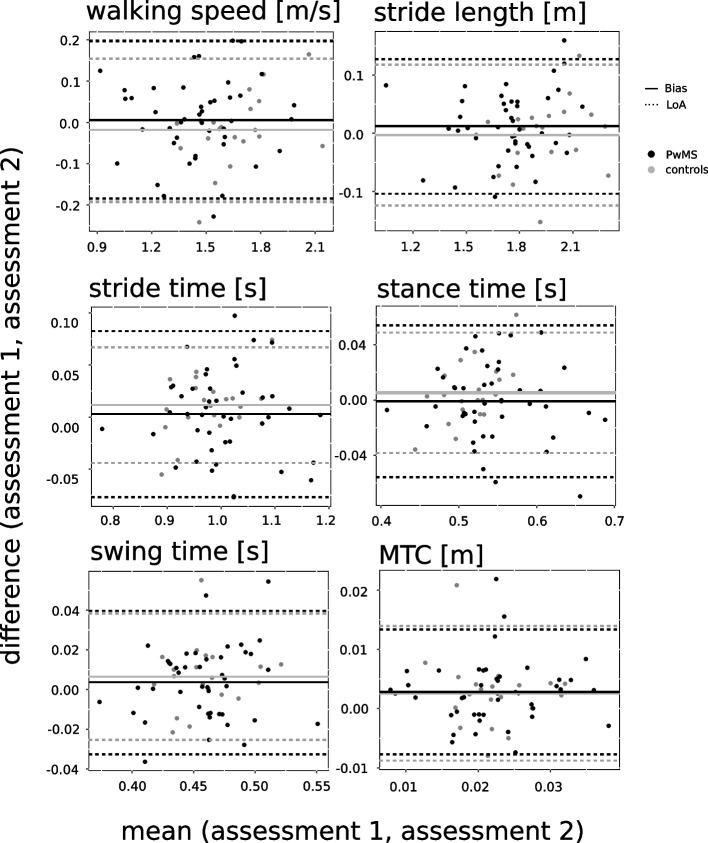
Bland–Altman plots for mean gait parameters show the difference versus the mean of both measurements for all single measurements (assessment 1, assessment 2). The solid line indicates the bias and the dashed lines the Limits of Agreement (95% confidence interval of the bias) for healthy controls (grey) and PwMS (black). MTC: minimum toe-to-floor distance

Table 2 summarizes the ICC values with 95% CI and the bias with 95% LoA of the mean gait parameters for healthy controls and PwMS as well as for both MS subgroups. For healthy controls, five out of six gait parameters showed excellent and only one (minimum toe-to-floor distance) fair ICC values (Table 2). For PwMS, all six gait parameters showed excellent ICC values (Table 2). In MS subgroups, the ICCs were good-to-excellent in MS subgroup 1 and excellent in MS subgroup 2. On average, MS subgroup 2 had higher ICC values than MS subgroup 1. Bland–Altman plots (Fig. 1) revealed no systematic bias for all mean gait parameters.

**Table 2:** Mean gait parameters of the 6-min walk

	**Assessment 1**	**Assessment 2**	ICC (95% CI; p-value)	Bias (lower – upper limit)	95% LoA
**Walking speed (m/s)**					
Control	1.67 ± 0.19	1.65 ± 0.23	0.91 (0.79–0.96; *p* < 0.001)	-0.018 (-0.192–0.155)	0.174
PwMS	1.46 ± 0.25*	1.46 ± 0.25*	0.93 (0.87–0.96; *p* < 0.001)	0.006 (-0.184–0.197)	0.191
MS subgroup 1	1.55 ± 0.14	1.57 ± 0.18	0.82 (0.59–0.92; *p* < 0.001)	0.026 (-0.167–0.219)	0.193
MS subgroup 2	1.38 ± 0.30*	1.37 ± 0.27*	0.94 (0.88–0.98; *p* < 0.001)	-0.010 (-0.197–0.177)	0.187
**Stride length (m)**					
Control	1.62 ± 0.17	1.61 ± 0.18	0.94 (0.85–0.98; *p* < 0.001)	-0.004 (-0.124–0.117)	0.121
PwMS	1.45 ± 0.19*	1.46 ± 0.20*	0.95 (0.92–0.97; *p* < 0.001)	0.012 (-0.104–0.128)	0.116
MS subgroup 1	1.53 ± 0.11	1.55 ± 0.14	0.86 (0.67–0.94; *p* < 0.001)	0.023 (-0.111–0.157)	0.134
MS subgroup 2	1.38 ± 0.21*	1.38 ± 0.21*	0.97 (0.94–0.99; *p* < 0.001)	0.002 (-0.095–0.099)	0.097
**Stride time (s)**					
Control	0.97 ± 0.05	0.98 ± 0.07	0.89 (0.74–0.96; *p* < 0.001)	0.011 (-0.044–0.067)	0.056
PwMS	1.00 ± 0.09	1.01 ± 0.08	0.88 (0.80–0.94; *p* < 0.001)	0.003 (-0.077–0.082)	0.080
MS subgroup 1	0.99 ± 0.07	0.99 ± 0.07	0.86 (0.68–0.94; *p* < 0.001)	-0.000 (-0.074–0.073)	0.074
MS subgroup 2	1.01 ± 0.10	1.02 ± 0.09	0.89 (0.77–0.95; *p* < 0.001)	0.005 (-0.080–0.091)	0.086
**Stance phase (s)**					
Control	0.51 ± 0.03	0.52 ± 0.04	0.82 (0.59–0.92; *p* < 0.001)	0.005 (-0.038–0.049)	0.043
PwMS	0.55 ± 0.06	0.55 ± 0.06	0.89 (0.80–0.94; *p* < 0.001)	-0.001 (-0.056–0.054)	0.055
MS subgroup 1	0.53 ± 0.04	0.53 ± 0.04	0.80 (0.57–0.92; *p* < 0.001)	-0.003 (-0.057–0.051)	0.054
MS subgroup 2	0.56 ± 0.07*	0.56 ± 0.06	0.91 (0.79–0.96; *p* < 0.001)	0.001 (-0.056–0.058)	0.057
**Swing phase (s)**					
Control	0.46 ± 0.03	0.46 ± 0.03	0.84 (0.64–0.93; *p* < 0.001)	0.007 (-0.026–0.039)	0.032
PwMS	0.46 ± 0.03	0.46 ± 0.04	0.87 (0.77–0.92; *p* < 0.001)	0.004 (-0.033–0.040)	0.036
MS subgroup 1	0.46 ± 0.03	0.47 ± 0.03	0.87 (0.71–0.95; *p* < 0.001)	0.003 (-0.027–0.032)	0.030
MS subgroup 2	0.45 ± 0.04	0.46 ± 0.04	0.86 (0.70–0.94; *p* < 0.001)	0.005 (-0.037–0.046)	0.041
**MTC (cm)**					
Control	2.09 ± 0.64	2.35 ± 0.54	0.52 (0.11–0.78; *p* = 0.008)	0.003 (-0.009–0.014)	0.011
PwMS	2.07 ± 0.74	2.36 ± 0.78	0.75 (0.58–0.85; *p* < 0.001)	0.003 (-0.008–0.013)	0.011
MS subgroup 1	2.10 ± 0.67	2.49 ± 0.77	0.70 (0.38–0.87; *p* < 0.001)	0.004 (-0.007–0.015)	0.011
MS subgroup 2	2.05 ± 0.81	2.24 ± 0.77	0.79 (0.58–0.90; *p* < 0.001)	0.002 (-0.008–0.012)	0.010

All values are expressed as mean ± standard deviation. Significant differences from healthy controls are indicated with ‘*’ (*p* < 0.05). MTC: minimum toe-to-floor distance

### Stability of gait variability parameters

The ICC values with 95% CI and the bias with 95% LoA of gait variability parameters for healthy controls and PwMS as well as for both MS subgroups were summarized in Table S1. For healthy controls, two out of six gait variability parameters showed good, one fair and three poor ICC values (Table S1). For PwMS, two out of six gait variability parameters showed good and four fair ICC values. In MS subgroups, the ICCs were poor-to-fair in MS subgroup 1 and fair-to-excellent in MS subgroup 2. On average, MS subgroup 2 had higher ICC values than MS subgroup 1. Bland–Altman plots (Figure S1) revealed no systematic bias for all gait variability parameters.

### Stability of observer rater and Timed-up and Go Test

**Fig. 2:**
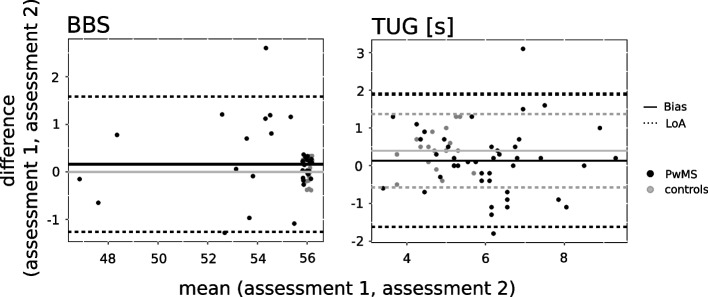
Bland–Altman plots for observer-rater test (Berg Balance Scale, BBS) and Timed-up and Go Test (TUG) show the difference versus the mean of both measurements for all single measurements (assessment 1, assessment 2). The solid line indicates the bias and the dashed lines the Limits of Agreement (95% confidence interval of the bias) for healthy controls (grey) and PwMS (black)

Concerning observer-rater test (BBS) and TUG, the ICC of the TUG was good for healthy controls and MS subgroup 2 and excellent for PwMS and MS subgroup 1 (Table 3). The ICC of the BBS was excellent for PwMS and both MS subgroups (Table 3). For healthy controls, the ICC was not calculated due to zero variance. Bland–Altman plots (Fig. 2) and analysis (Table 3) showed a bias between 0.079 (MS subgroup 2) and 0.4 (healthy controls) for TUG and a bias between 0.065 (MS subgroup 1) and 0.238 (MS subgroup 2) for BBS.

**Table 3:** Mean values of observer-rater test (BBS) and Timed-up and Go Test (TUG)

	**Assessment 1**	**Assessment 2**	ICC (95% CI, *p*-value)	Bias (lower – upper limit)	95% LoA
**BBS**					
Control	56.0 ± 0.0	56.0 ± 0.0	n.a	n.a	n.a
PwMS	54.3 ± 3.0	54.8 ± 2.4	0.95 (0.91–0.98; *p* < 0.001)	0.162 (-1.263–1.587)	1.425
MS subgroup 1	55.9 ± 0.4	55.9 ± 0.3	0.80 (0.52–0.93; *p* < 0.001)	0.065 (-0.428–0.553)	0.049
MS subgroup 2	53.0 ± 3.6*	53.9 ± 2.9	0.95 (0.87–0.98; *p* < 0.001)	0.238 (-1.611–2.088)	1.850
**TUG (s)**					
Control	4.51 ± 0.53	4.91 ± 0.66	0.66 (0.32–0.85; *p* = 0.001)	0.400 (-0.569–1.369)	0.069
PwMS	6.01 ± 1.39*	6.14 ± 1.37*	0.79 (0.64–0.88; *p* < 0.001)	0.134 (-1.630–1.898)	1.764
MS subgroup 1	5.31 ± 1.11	5.52 ± 0.96	0.83 (0.62–0.93; *p* < 0.001)	0.200 (-0.978–1.378)	1.178
MS subgroup 2	6.59 ± 1.36*	6.66 ± 1.45*	0.70 (0.41–0.86; *p* < 0.001)	0.079 (-2.076–2.234)	2.155

All values are expressed as mean ± standard deviation. Significant differences from healthy controls are indicated with ‘*’ (*p* < 0.05)

## Discussion

### Stability of sensor-based gait parameters reassessed after one year

The primary aim of the current study was to determine the stability of several gait parameters obtained by means of wearable inertial sensors (i.e. walking speed, stride length, stride time, the duration of the stance and swing phase as well as the minimum toe-to-floor distance) during a 6-min walk after one year in PwMS. We found that even after 12 months all mean gait parameters were stable in PwMS (ICC between 0.75 and 0.95; Table 2) and that the ICC values were close to the previously reported ICC values within one or 7–14 days measured by a stop-watch [3], an electronic walkway [15] or wearable inertial sensor [11, 12]. It is noteworthy that the interval between assessements in the current work was considerable longer than in the studies mentioned above. One may suggest that such a long time period of 12 months involves increasing unsystematic variance in the measures obtained. For example, it is conceivable that some PwMS might engage in health behavior, such as sports or physical therapy, in order to maintain their walking capacity, while other might neglect such options. Despite the fact that throughout the long period in the current work, such effects may yield considerable unsystematic variance, ICCs obtained were relatively similar to those of studies with short intervals. This is generally supportive of the methods implemented in the current work and suggests that intertial sensors provide reliable information even throughout longer time periods. Therefore, these sensor-based gait parameters can be identified as clinically relevant markers to evaluate treatment effects over a longer period of time (e.g. the effect of mindfulness training [24] or a six-month Tai Chi training on balance and coordination in PwMS; [2]). Looking at the MS subgroups, the ICCs were slightly higher in MS subgroup 2 (EDSS 2.0–5.0) than those in MS subgroup 1 (EDSS 0.0–1.5) and healthy controls. This is compatible with a previous study that showed that the higher the EDSS score, the more the reliability was increased [11].

It has been known that wearable inertial sensors are highly sensitive in the detection of gait disturbances, even in PwMS with lower or even no obvious gait impairment (e.g., [9, 12, 25, 26]). For example, during a 6-min walk mean gait parameters (mainly walking speed and stride length) consistently revealed significant differences between healthy participants and PwMS from as early as an EDSS value of 1.5 onwards (Table 2; [9]). Due to the good sensitivity of several mean gait parameters (i.e. walking speed and stride length) they can be identified as clinically relevant markers to assess gait impairment in PwMS.

Compared to the mean gait parameters, the parameters of gait variability (SD of gait parameters) showed only good-to-fair ICC values in PwMS (Table S1). However, looking at the MS subgroups the ICCs were slightly higher in MS subgroup 2 (EDSS 2.0–5.0) than those in MS subgroup 1 (EDSS 0.0–1.5). In addition, gait variability seems to be less sensitive in the detection of gait disturbances in early MS (e.g., [4, 27]. For example, Kalron [4] observed no significant differences in gait variability between PwMS classified in the lower disability level categories (EDSS 0 to 3.5) and a significant increase in gait variability when PwMS reached the moderate disability level, represented by EDSS scores of 4 and 5. Thus, parameters of gait variability seem to be less suitable for the detection of gait disturbances in early MS.

Concerning observer-rater test (BBS) and TUG, the ICC values after a period of one year were close to the ICC values of the measured mean gait parameters (Table 3) and in line with previous studies [28, 29]. However, both TUG and BBS showed a bias between 0.079 (MS subgroup 2) and 0.4 (healthy controls) and between 0.065 (MS subgroup 1) and 0.238 (MS subgroup 2), respectively. The time to complete the TUG (from signal to start to the moment the participant returns to the seat) was measured by a stop-watch. Thus, the bias in TUG can be caused by the subject who measures the time. Compared to that, automatically applicable gait assessments (as provided by wearable inertial sensors) provide more objective results. Nevertheless, while the TUG seems to be sensitive in the detection of gait disturbances in early MS (even depending on the subject who measures the time), the BBS seems not to be efficient in discrimination groups in early MS (as expected).

### Limitations of the study

Some limitations of the present study require consideration. First, no people with Primary Progressive MS (PPMS) participated in our study. Compared to the included PwMS, it is to be expected that gait parameters in PPMS will deteriorate over a longer period of time and thus, reveal a systematic bias for gait parameters. Second, the mean age for MS subgroup 2 was almost 10 years higher than the other groups (Table 1). Since gait parameters (e.g. walking speed or stride length) change with age [30], some of the differences between MS subgroup 2 and healthy controls can be explained by age-related effects. However, there is no effect of age on the ICC analysis. Third, differences between PwMS and healthy controls (Table 2) are not as noticeable as shown in previous studies (e.g., [9]). This effect can be attributed to the comparatively lower number of participants.

## Conclusion

The purpose of the current study was to examine multiple gait parameters (obtained by means of wearable inertial sensors) during a 6-min walk and to determine their stability after one year in PwMS. We found that even after one year all mean gait parameters showed excellent ICC values in PwMS. Due to the excellent stability of mean gait parameters after one year, these sensor-based gait parameters can be identified as clinically relevant markers to evaluate treatment effects over a longer period of time.

## Supplementary Information

**Additional file 1:** **Table S1. **Variability (SD) values of the 6-min walk. **Figure S1. **Bland-Altman plots for gait parameters variability show the differenceversus the mean of both measurements for all single measurements (assessment 1,assessment 2). The solid line indicates the bias and the dashed lines theLimits of Agreement (95% confidence interval of the bias) for healthy controls (grey)and PwMS (black). MTC: minimum toe-to-floor distance.

## Data Availability

The datasets used and/or analysed during the current study are available from the corresponding author on reasonable request.
