# Supplementary material for: Stability of sensor-based gait parameters reassessed after a period of one year in people with multiple sclerosis
Source: BMC Neurol. 2023 Mar 24;23:120. doi: 10.1186/s12883-023-03168-9 (PMC10037853; doi:10.1186/s12883-023-03168-9)
Supplement: Supplementary file 1 — Additional file 1: Table S1. Variability (SD) values of the 6-min walk. Figure S1. Bland-Altman plots for gait parameters variability show the differenceversus the mean of both measurements for all single measurements (assessment 1,assessment 2). The solid line indicates the bias and the dashed lines theLimits of Agreement (95% confidence interval of the bias) for healthy controls (grey)and PwMS (black). MTC: minimum toe-to-floor distance. [file 12883_2023_3168_MOESM1_ESM.docx]

**Supplementary Materials**

**Table S1.** Variability (SD) values of the 6-min walk.

|  | **assessment 1** | **assessment 2** | ICC (95% CI; p-value) | Bias (lower – upper limit) | 95% LoA |
| --- | --- | --- | --- | --- | --- |
| **velocity (m/s)** | | | | | |
| Control | 0.07 ± 0.02 | 0.06 ± 0.02 | 0.16 (-0.29-0.55; p=0.244) | -0.007 (-0.052-0.039) | 0.045 |
| PwMS | 0.07 ± 0.02 | 0.06 ± 0.02 | 0.55 (0.30-0.72; p=0.000) | -0.014 (-0.052-0.024) | 0.038 |
| MS subgroup 1 | 0.08 ± 0.02 | 0.06 ± 0.02 | 0.44 (0.01-0.73; p=0.023) | -0.016 (-0.056-0.024) | 0.040 |
| MS subgroup 2 | 0.07 ± 0.03 | 0.06 ± 0.02 | 0.60 (0.27-0.81; p=0.001) | -0.012 (-0.049-0.024) | 0.037 |
| **stride length (m)** | | | | | |
| Control | 0.05 ± 0.01 | 0.04 ± 0.01 | 0.26 (-0.19-0.63; p=0.124) | -0.006 (-0.031-0.019) | 0.025 |
| PwMS | 0.05 ± 0.01 | 0.04 ± 0.01 | 0.48 (0.22-0.68; p=0.000) | -0.009 (-0.033-0.015) | 0.024 |
| MS subgroup 1 | 0.05 ± 0.01 | 0.04 ± 0.01 | 0.45 (0.02-0.74; p=0.021) | -0.012 (-0.034-0.010) | 0.022 |
| MS subgroup 2 | 0.05 ± 0.01 | 0.04 ± 0.01 | 0.53 (0.17-0.76; p=0.003) | -0.006 (-0.031-0.018) | 0.025 |
| **stride time (s)** | | | | | |
| Control | 0.02 ± 0.00 | 0.02 ± 0.01 | 0.49 (0.07-0.76; p=0.013) | -0.000 (-0.011-0.010) | 0.011 |
| PwMS | 0.03 ± 0.01 | 0.02 ± 0.01 | 0.71 (0.52-0.83; p=0.000) | -0.003 (-0.019-0.014) | 0.016 |
| MS subgroup 1 | 0.02 ± 0.01 | 0.02 ± 0.01 | 0.39 (-0.49-0.71; p=0.039) | -0.002 (-0.015-0.011) | 0.013 |
| MS subgroup 2 | 0.03 ± 0.02 | 0.02 ± 0.01 | 0.75 (0.49-0.88; p=0.000) | -0.003 (-0.022-0.016) | 0.019 |
| **stance phase (s)** | | | |  | |
| Control | 0.01 ± 0.00 | 0.01 ± 0.00 | 0.60 (0.22-0.82; p=0.002) | -0.002 (-0.009-0.004) | 0.007 |
| PwMS | 0.02 ± 0.01 | 0.02 ± 0.01 | 0.62 (0.40-0.77; p=0.000) | -0.004 (-0.018-0.011) | 0.014 |
| MS subgroup 1 | 0.02 ± 0.01 | 0.01 ± 0.00 | 0.38 (-0.06-0.70; p=0.045) | -0.003 (-0.013-0.007) | 0.010 |
| MS subgroup 2 | 0.02 ± 0.01 | 0.02 ± 0.01 | 0.64 (0.33-0.83; p=0.000) | -0.004 (-0.021-0.013) | 0.017 |
| **swing phase (s)** | | | | | |
| Control | 0.01 ± 0.00 | 0.01 ± 0.00 | 0.74 (0.45-0.89; p=0.000) | -0.001 (-0.006-0.003) | 0.004 |
| PwMS | 0.01 ± 0.00 | 0.01 ± 0.00* | 0.59 (0.35-0.75; p=0.000) | -0.001 (-0.008-0.007) | 0.008 |
| MS subgroup 1 | 0.01 ± 0.00 | 0.01 ± 0.00 | 0.39 (-0.06-0.70; p=0.042) | -0.000 (-0.006-0.005) | 0.006 |
| MS subgroup 2 | 0.01 ± 0.00 | 0.01 ± 0.01* | 0.58 (0.24-0.80; p=0.001) | -0.001 (-0.010-0.008) | 0.009 |
| **MTC (cm)** | | | | | |
| Control | 0.46 ± 0.11 | 0.48 ± 0.09 | 0.33 (-0.12-0.67; p=0.071) | 0.000 (-0.002-0.002) | 0.002 |
| PwMS | 0.44 ± 0.12 | 0.45 ± 0.11 | 0.52 (0.27-0.71; p=0.000) | 0.000 (-0.002-0.002) | 0.002 |
| MS subgroup 1 | 0.44 ± 0.09 | 0.47 ± 0.10 | 0.17 (-0.29-0.56; p=0.238) | 0.000 (-0.002-0.003) | 0.002 |
| MS subgroup 2 | 0.44 ± 0.15 | 0.43 ± 0.11 | 0.71 (0.44-0.86; p=0.000) | -0.000 (-0.002-0.002) | 0.002 |

All values are expressed as mean ± standard deviation. MTC minimum toe-to-floor distance. Significant differences from healthy controls are indicated with ‘*’ (p<0.05).


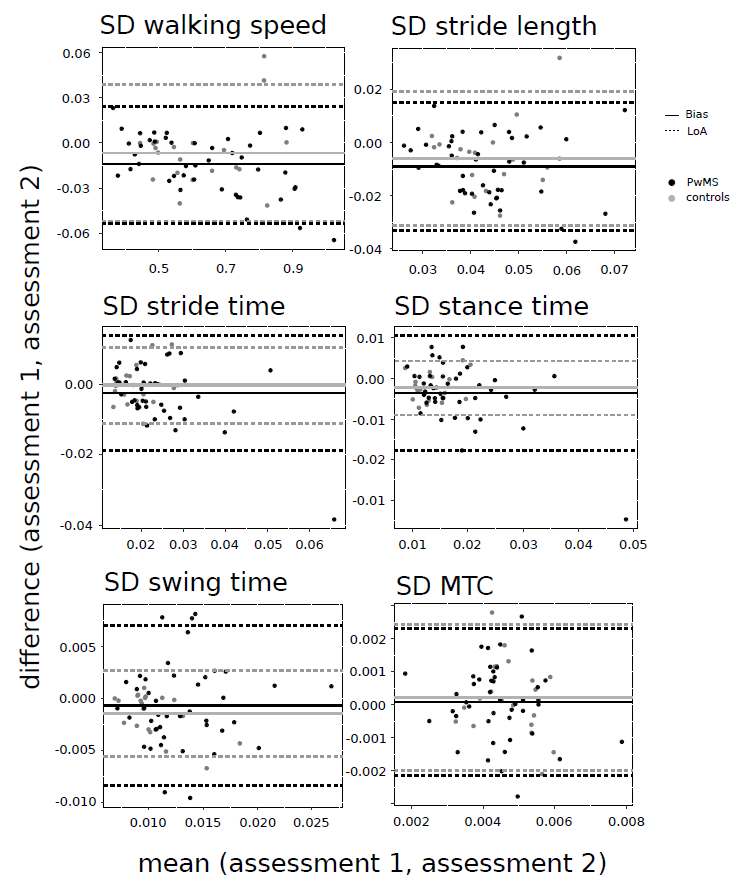


**Figure S1:** Bland-Altman plots for gait parameters variability show the difference versus the mean of both measurements for all single measurements (assessment 1, assessment 2). The solid line indicates the bias and the dashed lines the Limits of Agreement (95% confidence interval of the bias) for healthy controls (grey) and PwMS (black). MTC: minimum toe-to-floor distance.
